# Effects of propofol on early and late cytokines in lipopolysaccharide-induced septic shock in rats

**DOI:** 10.1016/S1674-8301(10)60052-8

**Published:** 2010-09

**Authors:** Sha Li, Hongguang Bao, Liu Han, Lele Liu

**Affiliations:** Department of Anesthesiology, Nanjing First Hospital Affiliated to Nanjing Medical University, Nanjing 210006, Jiangsu Province, China

**Keywords:** propofol, sepsis, tumor necrosis factor-α, interleukin-6, high mobility group box 1

## Abstract

**Objective:**

It has been reported that the intravenous anesthetic propofol (PPF) has anti-inflammatory effects *in vitro* and in patients. The purpose of this study was to investigate whether PPF has anti-inflammatory effects in lipopolysaccharide (LPS)-induced septic shock by inhibiting the induction of pro-inflammatory cytokines [interleukin-6 (IL-6) and tumor necrosis factor-α (TNF-α)] and high mobility group box 1 (HMGB1) in rats.

**Methods:**

Thirty six male Wistar rats were randomly assigned to one of three groups (control group, PPF + LPS group and LPS group; *n* = 12 per group). Control group rats received a 0.9% NaCl solution (NS) by the tail vein. The PPF + LPS group rats received PPF (10 mg/kg bolus, followed by infusion at 10 mg/(kg·h) through a femoral vein catheter) 1 h before LPS (7.5 mg/kg) administration *via* the tail vein. The LPS group rats received injection of LPS (7.5 mg/kg) *via* the tail vein. Hemodynamic effects were recorded as well as mortality rates, and plasma cytokine con-centrations (TNF-α, IL-6, HMGB1) were measured for the 24-h observation period.

**Results:**

The mean arterial pressure and heart rate of the PPF + LPS group were more stable than those of the LPS group. The mortality at 24 h after the administration of the LPS injection was much higher in the LPS group (58.3%) compared to the PPF + LPS group (25.0%). Plasma concentrations of cytokines (IL-6 and TNF-α) and HMGB1 were significantly reduced in the PPF + LPS group compared to the LPS group (*P* < 0.05).

**Conclusion:**

Pretreatment with PPF reduced the mortality rate of rats and attenuated the pro-inflammatory cytokine responses in an endotoxin shock model through an anti-inflammatory action inhibiting induction of HMGB1.

## INTRODUCTION

Sepsis and septic shock are common problems in the intensive care unit and carry a very high mortality rate. Various complications that accompany sepsis, such as disseminated intravascular coagulation (DIC) and acute respiratory distress syndrome (ARDS), are refractory to therapy, even with current advanced therapeutic methods. Sepsis is potentially fatal if not treated appropriately[1],[2], and many clinical therapies, including anti-inflammatory strategies and fluid replacement, have been used in recent years for treatment of sepsis[3],[4]. However, the effectiveness of these strategies is largely unproven, and the morbidity and mortality associated with sepsis are still increasing. As a consequence, it is vital that we find new interventions to treat sepsis.

It has been demonstrated that high mobility group box 1 (HMGB1) protein is an important late-phase mediator in the pathogenesis of sepsis[5]. HMGB1 is a non-histone nuclear protein originally identified as an important factor in the regulation of genetic information by facilitating the binding of transcription factors to their cognate DNA sequences[6]. Wang *et al.* found that HMGB1 could be actively released from necrotic or damaged cells, or secreted by activated monocytes/macrophages. It also could bind to the receptor for advanced glycation end-products (RAGE), allowing transduction of the signal[7]. Moreover, the release of HMGB1 into the extracellular space is controlled by pro-inflammatory cytokines[8].

Propofol (PPF) is a sedative-hypnotic intravenous anesthetic drug, which is widely used in the intensive care unit as it can be easily titrated and offers the prospect of rapid recovery. When used for sedation or induction and maintenance of anesthesia, PPF has been shown to have a number of hemodynamic effects, including substantial reductions in systemic vascular resistance, heart rate, stroke volume and cardiac output[9],[10]. When PPF was used for induction and maintenance of anesthesia in septic sheep, the hemodynamic parameters were reduced compared to those in non-anesthetized septic sheep[11]. Recently, several investigators have reported that PPF inhibited the production of lipopolysaccharide (LPS)-induced tumor necrosis factor-α (TNF-α) and other pro-inflammatory cytokines in endotoxemic rats[12],[13]. However, the relationship between PPF and HMGB1 has not yet been determined. We hypothesized that PPF could reduce the levels of pro-inflammatory cytokines and HMGB1 in serum, which in turn could prevent LPS-induced inflammatory responses. To test this hypothesis, we examined the effects of PPF on LPS-induced sepsis in a rat model in this study.

## MATERIALS AND METHODS

### Animals and materials

Thirty-six male Wistar rats, weighing (315±50) g, were tested in this study. The experimental protocol was approved by the Animal Care Committee of Nanjing Medical University, and the care and handling of the animals were in accordance with National Institutes of Health guidelines. All animals were provided with clean food and water. LPS used in this study was derived from *Escherichia coli* endotoxin (serotype: O111:B4; Sigma, USA) and dissolved in sterile saline. PPF (AstraZeneca, Italy) was a 1% solution and EDTA-free.

### Experimental protocols

The animals were randomly assigned to three groups. All of the rats were anesthetized with an intraperitoneal injection of 10% chloral hydrate (0.4 mL/100g). Control group: rats received 0.9% NaCl [10 mg/kg bolus, followed by infusion at 10 mg/(kg·h)] through the left femoral vein cannula 1 h before intravenous administration of 0.9% NaCl into the tail vein (7.5 mg/kg). PPF+LPS group: PPF [10 mg/kg bolus, followed by infusion at 10 mg/(kg·h)] was infused continuously through the left femoral vein cannula 1 h before injection of LPS (7.5 mg/kg) into the tail vein. LPS group: rats received an intravenous injection of 0.9% NaCl through the left femoral vein cannula 1 h before injection of LPS (7.5 mg/kg) into the tail vein. The right femoral artery was cannulated to monitor mean arterial pressure (MAP) and heart rates (HR). The mortality of rats in each group was determined at 24 h after LPS injection.

### Measurement of plasma cytokine levels

At 4, 8, 12 and 24 h after administration of LPS, whole blood was drawn from the right femoral artery (1.0 mL each). The plasma was immediately separated by centrifugation at 1,800 *g* for 15 min at 4°C, and then divided into aliquots and stored at -20°C until assayed. The levels of pro-inflammatory cytokines [interleukin 6 (IL-6) and TNF-α] and HMGB1 in plasma were quantified using enzyme-linked immunosorbent assays (ELISA) kits (American R&D and Bender Medsystems, USA).

### Statistical analysis

The data were presented as mean±SD. Analysis of variance (ANOVA) was used to evaluate the differences between groups at the various time points, and two-way analysis of variance for repeated measurements with multiple comparisons (Bonferroni *t*). A *P* value of less than 0.05 was considered statistically significant. Statistical analyses were performed using the SPSS 16.0 software package.

## RESULTS

### Hemodynamic parameters and mortality rates

**Fig. 1:**
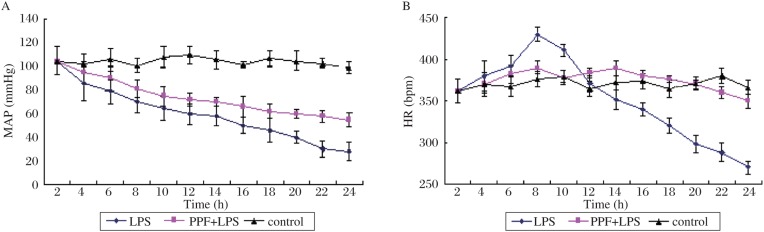
MAP (*A*) and HR (*B*) at baseline and after LPS or saline injection in different groups. MAP: mean arterial pressure; HR: heart rates; LPS: lipopolysaccharide.

No significant differences were noted in baseline HR or MAP among the groups (***Fig. 1***). An endotoxin injection reduced MAP in both the LPS group and PPF+LPS group, while MAP remained unchanged in the control group. At 24 h after the administration of LPS, MAP decreased by 5.0%, 72.6% and 43.5% in the control group, LPS group and PPF+LPS group, respectively. There were significant differences in MAP between the LPS group and PPF + LPS group at 16, 18, 20, 22 and 24 h time points (*P* < 0.05).

There were no significant differences in HR among the groups for the first 6 h of the experiment (***Fig. 1***). In the LPS group, HR significantly increased at 8 h and then gradually decreased. This decrease was statistically significant by 16 h. Thus, there were significant differences in HR at 8, 16, 18, 20, 22 and 24 h time points between the LPS group and PPF+LPS group (*P* < 0.05).

**Fig. 2:**
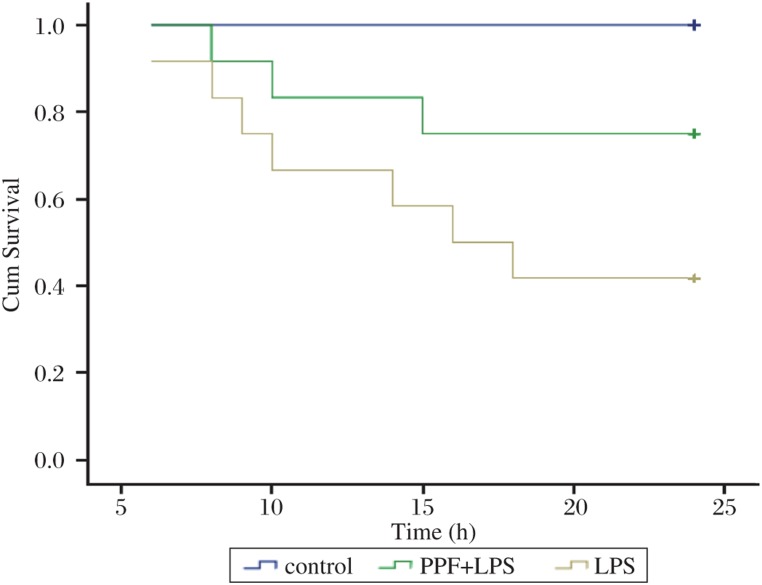
Survival curves for LPS, PPF+LPS and control groups. LPS: lipopolysaccharide; PPF: propofol.

Rat survival was analyzed at 24 h after the surgical procedures, and mortality after endotoxin injection was 58.3%, 25.0% and 0% for the LPS, PPF + LPS and the control group, respectively (***Fig. 2***). The mortality in the PPF pretreatment group (PPF+LPS) group was significantly lower than that in the LPS group (*P* < 0.01).

### Plasma cytokine concentrations

Plasma level of IL-6 increased in the LPS group, while pretreatment with PPF significantly decreased IL-6 level. At 4, 8 and 12 h after LPS administration, the serum levels of IL-6 were significantly lower in the PPF+ LPS group than those in the LPS group (*P* < 0.05, see ***Table 1***). Plasma level of TNF-α also increased after LPS treatment. Serum TNF-α level was significantly lower in the PPF+LPS group compared to the LPS group at 4 h after LPS administration (*P* < 0.05, see ***Table 2***). Plasma HMGB1 level increased over time after LPS administration in the LPS and PPF+LPS group. Pretreatment with PPF resulted in lower HMGB1 level at 8, 12 and 24 h after LPS administration compared to the LPS group (*P* < 0.05, see ***Table 3***).

**Table 1:** Plasma IL-6 concentration

Group	Time (h)			
	4	8	12	24
Control	43.65 ± 6.28*	52.29 ± 4.93*	45.51 ± 5.73*	47.12 ± 7.18
LPS	1192.68 ± 207.50^#^	812.43 ± 107.62^#^	595.86 ± 94.46^#^	48.03 ± 5.62
PPF + LPS	743.72 ± 103.68*^#^	582.63 ± 60.36*^#^	320.42 ± 56.18*^#^	47.23 ± 4.57

*Compered with the LPS group (*P* < 0.05); *Compered with the control group (*P* <0.05). LPS: lipopolysaccharide; PPF: propofol.

(pg/mL, *n* = 12)

**Table 2:** Plasma TNF-α concentration

Group	Time (h)			
	4	8	12	24
Control	25.96 ± 2.28*	26.29 ± 1.95*	25.51 ± 1.05	25.12 ± 1.30
LPS	61.08 ± 3.77^#^	36.38 ± 1.89^#^	27.09 ± 2.13	24.98 ± 1.27
PPF + LPS	40.85 ± 3.89*^#^	31.47 ± 4.14*^#^	26.96 ±1.02	25.29 ± 1.23

*Compered with the LPS group (*P* < 0.05); ^#^Compered with the control group (*P* < 0.05). LPS: lipopolysaccharide; PPF: propofol.

(pg/mL, *n* = 12)

**Table 3:** Plasma HMGB1 concentration

Group	Time (h)			
	4	8	12	24
Control	5.72 ±1.13	6.08 ± 1.88*	5.96 ± 0.97	9.23 ± 1.37*
LPS	5.80 ± 1.34	10.21 ± 2.26^#^	12.41 ± 2.03	14.58 ± 3.67^#^
PPF + LPS	5.79 ± 1.45	8.35 ± 1.07*^#^	9.05 ± 1.29	9.65 ± 3.95*^#^

*Compered with the LPS group (*P* < 0.05); ^#^Compered with the control group (*P* < 0.05). LPS: lipopolysaccharide; PPF: propofol.

(µg/mL, *n* = 12)

## DISCUSSION

Injection of endotoxin alone is the most common method to duplicate septic shock in rats. Previous studies have shown that PPF inhibited inflammatory responses during endotoxemia and sepsis[12],[13]. PPF can also attenuate the base deficit and activation of neutrophils in endotoxemia, which suggest that PPF may prevent the development of metabolic acidosis during endotoxemia[14]. However, there were few reports about the effect of PPF on mortality in response to endotoxin-induced shock, especially within 24 h after an endotoxin injection. The study presented here clearly demonstrated that pretreatment with PPF reduced the mortality of rats at 24 h after endotoxin injection.

PPF is a popular intravenous anesthetic, and is increasingly used in critically ill septic patients[15]. Several publications demonstrated that PPF has a beneficial effect in experimental models of septic shock[16],[17]. In our study, PPF administration inhibited development of hypotension, moderately regulated the HR and suppressed the levels of pro-inflammatory cytokines, and the results were in accordance with a previous study[18]. Cytokines have been implicated as important factors in the pathophysiology of endotoxic shock and the development of cardiovascular dysfunction in endotoxemia[19],[20]. Therefore, we measured plasma cytokine concentrations to investigate the mechanism by which PPF could reduce mortality in endotoxic shock. We found that endotoxin not only provoked the elevation of pro-inflammatory cytokines such as TNF-α and IL-6 but also the late cytokine--HMGB1 concentration in the plasma in septic rats. By contrast, in the presence of PPF administration, there was an inhibitory effect on endotoxin-induced production of pro-inflammatory cytokines and HMGB1.

Since TNF-α is considered to play a key role in the pathogenesis of sepsis and septic shock, TNF-α is an important component of the pro-inflammatory cytokines during the early stages of septic shock. Therefore, we measured the plasma TNF-α level in our study. Previously, measurement of IL-6 level enabled us to accurately predict individuals who were at significant risk of sepsis[21], so we also measured IL-6 concentrations. It has recently been demonstrated that the HMGB1 protein is an important late-phase mediator in the pathogenesis of sepsis[7]. Anti-HMGB1 treatment, with either antibodies, specific antagonists or other pharmacological agents, is beneficial in many preclinical inflammatory disease models, and alleviates the severity of such diseases and reduces their mortality[22]. We found that the plasma levels of TNF-α and IL-6 in LPS-induced septic rats were suppressed by PPF. Moreover, PPF also suppressed the plasma level of HMGB1. However, we did not measure *HMGB1* mRNA expression, which may be a limitation of this study.

Wang *et al.*[7] reported that LPS, TNF-α or IL-1 can stimulate the production of HMGB-1 by monocytes. They also found that there was a reciprocal functional relationship between the activities of the early (TNF-α and IL-1) and late (HMGB-1) cytokines[23], which apparently meant that HMGB-1 could participate in the “cross-talk” for the propagation and amplification of downstream pro-inflammatory responses. In our study, we found that the changes in the plasma concentrations of TNF-α, IL-6 and HMGB1 were always consistent in both the PPF+LPS group and LPS group, which was in agreement with the literature[7],[23]. The above data indicated that pro-inflammatory cytokines and HMGB1 contributed to the effects of sepsis.

Some important questions remain unanswered: what are the actual mechanisms responsible for the hemodynamic effects of PPF, and what is the mechanism by which PPF mediates its protective effect on pro-inflammatory cytokines and HMGB1. In a recent study, Song *et al.*[24] indicated PPF suppressed hepatic NF-κB activation, consequently inhibiting the transcription and translation of pro-inflammatory cytokines during polymicrobial sepsis. Whether PPF reduces the levels of HMGB1 in plasma through suppression of the NF-κB activation or by other signaling pathways remains to be further studied.

PPF was carried in a lipid emulsion in the present study. Several studies on the relationship between lipids and endotoxemia have been reported. Gordon *et al.*[25] found that patients who are critically ill from a variety of causes have extremely low cholesterol and lipoprotein concentrations. Correction of the hypolipidemia by a reconstituted high density lipoprotein preparation offers a new strategy for the prevention and treatment of endotoxemia. The ability of lipids to reduce cytokine production *in vitro* after endotoxin stimulation has been reported[25],[26]. In addition, infusions of reconstituted apolipoprotein A-I, chylomicrons or high-density lipoprotein have been shown to decrease TNF-α release and to attenuate shock in animal models[27]–[29]. However, Tobias *et al.*[30] reported that lipids may be less effective in neutralizing endotoxin in the acute phase. Our study did not investigate the relationship between lipids alone and endotoxemia, and further study of PPF, lipids alone and endotoxemia are required.

Pan *et al.*[31] found that chloral hydrate could significantly attenuate acute inflammation in mice treated with LPS/D-GalN and zymosan A. In this study, we also used chloral hydrate as the anesthetic, but the dose we used here was lower than that used by Pan *et al.*[31], and the rats were anesthetized by intraperitoneal injection, not by intravenous injection. Chloral hydrate has only anaesthetic and sedative effects on rats at the dose we used; therefore, the anti-inflammatory effect we found was induced by PPF but not by chloral hydrate.

Critically ill patients in sepsis/septic shock often suffer a high degree of stress due to pain and anxiety and the organ specific response to sepsis. Achieving an adequate level of sedation will enable the doctors to manage these patients. Our study suggests that PPF may be of benefit by preventing the inflammatory effects and sedating septic patients. Further investigations and clinical trial are needed.

In summary, the study presented here shows that pretreatment with PPF before LPS injection significantly reduced the mortality rate, and attenuated pro-inflammatory cytokine and HMGB1 responses in rats. These findings suggest that PPF may be beneficial for the treatment of sepsis.
